# Research Defence Society

**Published:** 1908-05-02

**Authors:** 


### RESEARCH DEFENCE SOCIETY.

(*To the Editor of* The Hospital.)

Sir,—A society has been formed, with the name of the Research Defence Society, to make known the facts as to experiments on animals in this country; the immense importance to the welfare of mankind of such experiments; and the great saving of human life and health directly atributable to them. The great advance that has been made during the last quarter of a century in our knowledge of the functions of the body, and of the causes of disease, would have been impossible without a combination of experiment and observation.

The use of antiseptics and the modern treatment of wounds is the direct outcome of the experiments of Pasteur and Lister. Pasteur's discovery of the microbial cause of puerperal fever has in itself enormously reduced the deaths of women in child-birth. The nature of tuberculosis is now known, and its incidence has materially diminished. We owe the invention of diphtheria anti-toxin entirely to experiments on animals. The causes of plague, cholera, typhoid, Mediterranean fever, and sleeping sickness, have been discovered solely by the experimental method. Not only have a large number of drugs been placed at our disposal, but accurate knowledge has replaced the empirical use of many of those previously known. The evidence before the Royal Commission has shown that these experiments are conducted with proper care; the small amount of pain or discomfort inflicted is insignificant compared with the great gain to knowledge and the direct advantage to humanity.

While acknowledging in general the utility of the experimental method, efforts have been made by a section of the public to throw discredit on all experiments involving the use of animals. The Research Defence Society will therefore endeavour to make it clear that medical and other scientific men who employ these methods are not less humane than the rest of their countrymen, who daily, though perhaps unconsciously, profit by them. The Society proposes to give information to all inquirers, to publish *précis*, articles, and leaflets, to make arrangements for lectures, to send speakers (if required) to debates, and to assist all who desire to examine the arguments on behalf of experiments on animals. It hopes to establish branches in our chief cities, and thus to be in touch with all parts of the kingdom; and to be at the service of municipal bodies, hospitals, and other public institutions.

The Society was formed on January 27 of the present year, and already numbers more than 800 members. It is not an association of men of science or of medical men alone; its membership has been drawn from all departments of public life, and includes representatives of every class of educated Englishmen and Englishwomen, including many who have taken an active part in the prevention of cruelty to animals. This fact is in itself a remarkable protest against the attacks which have been made on the researches that the Society has been formed to defend. The annual subscription is 5s. to cover working expenses; but larger subscriptions, or donations, will be gladly received. The acting Hon. Treasurer, *pro tem.*, is Mr. J. Luard Pattisson, C.B. (of the Lister Institute), and an account in the Society's name has been opened with Messrs. Coutts and Company, 440 Strand. The Hon. Secretary is Mr. Stephen Paget, 70 Harley Street, W., to whom all communications should be addressed.

Yours faithfully, Cromer, President.

The following is a list of the Vice-Presidents of the Society :—

The Duke of Abercorn, Sir T. Clifford Allbutt, Sir L. Alma-Tadema, R.A., Sir William Anson, M.P., Lord Avebury, the Marquis of Bath, Lord Blyth, the Dean of Canterbury, Earl Cathcart, Lord Robert Cecil, K.C., M.P., the Dean of Chester, Lord Cheylesmore (Chairman, Middlesex Hospital), the Dean of Christ Church, Sir James Crichton-Browne, Sir Savile Crossley, Sir Edmund Hay Currie, the Rev. Dr. Dallinger, Mr. Francis Darwin, Sir George H. Darwin, Sir James Dewar, Sir A. Conan Doyle, Canon Duckworth, the Bishop of Edinburgh, the Bishop of Exeter, the Rev. A. M. Fairbairn (Principal of Mansfield College, Oxford), Lord Faber, Lord Farrer, Sir Luke Fildes, R.A., Sir David Gill, the Bishop of Grantham, the Hon. Walter Guinness, M.P., the Earl of Halsbury, Mr. H. A. Harben (Chairman, St. Mary's Hospital), Mr. J. T. Helby (Chairman, Metropolitan Asylums Board), the Hon. Sydney Holland (Chairman, London Hospital), Sir Joseph Dalton Hooker, Sir William Huggins, Sir Alfred Jones, the Earl of Kilmorey (Chairman, Charing Cross Hospital), Sir E. Ray Lankester, Lord Lindley, Sir Norman Lockyer, the Right Hon. Walter H. Long, M.P., Mr. Henry Lucas (Chairman, University College Hospital), the Hon. G. W. Spencer Lyttelton, Mr. Frederick Macmillan (Chairman, National Hospital for the Paralysed and Epileptic), Sir Herbert E. Maxwell, Lord Methuen, the Earl of Northbrook (President, Cancer Hospital), Lord Northcliffe, Professor Osler, the Bishop of Oxford, Sir Gilbert Parker, M.P., Sir Frederick Pollock, Sir John Dickson-Poynder, M.P. (Chairman, Great Northern Hospital), the Bishop of North Queensland, Sir William Ramsay, Sir James Reid, the Dean of Ripon, Sir Henry Roscoe, the Earl of Rosse, Lord Rothschild, Sir Arthur Rücker, the Dean of Salisbury, the Marquis of Sligo, Sir Cecil Clementi Smith, Sir Thomas Smith, the Hon. W. F. D. Smith, M.P. (Chairman of Removal Fund, King's College Hospital), Sir Richard Solomon, Sir Edgar Speyer (President, Poplar Hospital), Lord Stanley, Lord Strathcona, Sir Frederick Treves, Sir John Batty Tuke, M.P., Mr. James G. Wainwright (Chairman, St. Thomas's Hospital), Earl Waldegrave, Mr. A. W. West (Treasurer and Chairman, St. George's Hospital), Sir James Whitehead (First President of the Lister Institute), Sir Samuel Wilks, Sir Alfred Wills, the Rev. H. G. Woods (Master of the Temple), and Lord Claud Hamilton.

